# Hand hygiene knowledge, perception, and self-reported performance among nurses in Kelantan, Malaysia: a cross-sectional study

**DOI:** 10.1186/s12912-022-00820-6

**Published:** 2022-01-31

**Authors:** Mohamad Hazni Abd Rahim, Mohd Ismail Ibrahim

**Affiliations:** Department of Community Medicine, School of Medical Sciences, Universiti Sains Malaysia, 16150 Kubang Kerian, Kota Bharu, Kelantan Malaysia

**Keywords:** Knowledge and Perception score, Self-reported hand hygiene performance, Nurses, Tertiary care, Kelantan

## Abstract

**Background:**

Nurses interact with patients 24 hours a day, and this connection has the potential to raise the risk of infection transmission to patients. Nursing plays a vital role in pre-venting healthcare-associated infections (HCAIs) by ensuring that hand hygiene (HH) practises are followed and maintained. The purpose of this study was to evaluate nurses’ knowledge, perceptions, and self-reported HH performance scores, as well as their correlation, in tertiary care hospitals in Kelantan, Malaysia.

**Methods:**

A cross-sectional study was undertaken in all four Kelantan tertiary care hospitals from December 2019 to February 2020. A stratified random selection method was used to obtain a sample of 438 registered nurses. A validated WHO self-administered HH knowledge and perception questionnaire for healthcare personnel was used to measure HH knowledge, perception, and self-reported HH performance.

**Results:**

The mean (SD) score of knowledge was 15.08 (1.96) out of the total 25. The score of perception participants towards HH was 68.02 (10.14) out of the total 81 and the average self-reported HH performance was 87.58 (12.03) out of 100. Pearson’s correlation analysis showed significant positive correlations between perception and knowledge scores; r (436) =0.17, *p*<0.001 and Perception and self-reported HH performance scores; r (436) =0.27, *p*<0.001.

**Conclusions:**

There is a strong link between knowledge and perception scores. Lack of understanding of HH during patient care might lead to a negative perception, which can affect overall self-reported HH performance. The need of monitoring and maintaining HH knowledge among nurses was established in this study.

## Background

Healthcare-associated infections (HCAIs) are estimated to affect hundreds of millions of patients globally per year, resulting in major mortality and financial losses for health services, according to the World Health Organization (WHO). In developed countries, 6 to 7 out of every 100 hospitalised patients will develop at least one HCAIs, while 10 in developing countries will [1]. Prevalence HCAIs were found in 13.9 out of 100 patients in Malaysia [2]. Longer hospital stays, long-term injury, microorganism tolerance, increased financial pressure, high costs, and premature deaths are all consequences of HCAIs. HCAIs can affect patients in any type of treatment setting and can even appear after they have been discharged. They are the most common adverse event in healthcare, and no agency or country will claim to have solved the problem. In Geneva 2005, the World Health Organization (WHO) initiated the first patient safety initiative and a multimodal hand hygiene improvement strategy in the hopes of strengthening public health action and reducing the number of people who die from HCAIs.

Hand hygiene (HH) before and after contact with a patient is the most effective strategy to prevent infection transmission [3]. The most prevalent mechanism for diseases connected with health care to spread is through the hands [4]. Increased hand hygiene performance in health care can lower HCAIs by up to 50% [5]. Nursing is important in health care because it involves patients of all ages receiving self-directed and integrated care in a variety of settings [6]. Nurses are one of the many multidisciplinary health care workers who engage with patients on a regular basis in bedside care. Nurses are the only health-care professionals who work with patients around the clock [7]. As a result, nursing plays an important role in preventing HCAIs by ensuring that correct hand hygiene guidelines are followed.

The WHO hand hygiene programme has been found to effectively promote hand hygiene compliance [8, 9]. Hand hygiene performance, perceptions, and knowledge must all be monitored as part of phase three of the WHO Guidelines’ HH Multimodal Improvement Strategy [10]. To achieve the Malaysian government’s National Patient Safety Goal, all stakeholders, including universities, are encouraged to conduct scientific research to fill gaps and expand the pool of patient safety information. The growing emphasis on reducing HCAIs in Malaysian hospitals emphasises the importance of improving treatment quality while maintaining patient safety concerns [11–14]

Hand hygiene promotion success necessitates not only the implementation of interventions, but also a better understanding of HCW perceptions of HCAIs and HH [15]. Individual characteristics such as behaviour and psychological determinants (knowledge, attitude, intentions, beliefs, and perceptions) may provide additional information on hand hygiene performance [16]. Hand hygiene knowledge, in particular, has been identified as a critical factor in improving compliance rates [17]. In recent findings [18], lack of awareness has been identified as a self-reported factor and barrier that may impact hand hygiene and flow compliance. It has also been demonstrated that nurses’ knowledge, perceptions, and attitudes toward HH influence their HH performance [19, 20]. In the local setting, there are activities relating to the issue, but only a handful of them result in publication. As a result, this study describes a survey that was undertaken in this place, and its addition to current knowledge may bring fresh insights into hand hygiene practises.

The objective of the current study is to examine the relationship between hand hygiene knowledge, perceptions, and self-reported performance among nurses in Kelantan, Malaysia. In addition, we wish to prove that when staff, particularly nurses, have a strong association between hand hygiene knowledge, perception, and self-reported performance, the risk of hospital acquired infection is lowered. Thus, the following are some of our research questions:

1. Is there a correlation between knowledge score and perception of self-performance?
2. Is there a correlation between perception scores and self-performance scores?

## Methods

### Study design and participants

A cross-sectional survey was done among registered nurses working in tertiary hospitals in Kelantan, Malaysia, from December 15, 2019 to February 15, 2020. Tertiary hospitals are specialized advisory centers that provide professionals and facilities for specialized diagnosis and treatment. The four tertiary hospitals were Hospital Raja Perempuan Zainab II (HRPZII), Hospital Universiti Sains Malaysia (HUSM), Hospital Sultan Ismail Petra (HSIP), and Hospital Tanah Merah (HTM). The total number of nurses in tertiary hospitals in Kelantan was 3366. Capacity of nurses each hospital in HRPZII (1465 nurses), HUSM (1382 nurses), HSIP (300 nurses) and HTM (219 nurse).

### Study tools

Two self-administered questionnaires were used to collect the data: the WHO hand hygiene knowledge questionnaire for healthcare workers (revised in 2009) and the WHO hand hygiene perception questionnaire for healthcare workers (revised in 2009) [21]. The participants were given a total of 35 questions to answer to. The surveys took an average of ten minutes to complete. The study’s three domains were established using both questionnaires: sociodemographic data (domain A), HH knowledge (domain B), and a perception survey (domain C).

Background information, age, gender, marital status, hospital, job characteristics, receive formal training, and education level were among the sixteen items in Domain A (from Q1 to Q16). Each respondent who scored with the correct answers in Domain B from Q17 to Q24 was counted and recorded.

The domain C questionnaire comprised 11 items (Q25-Q35), three of which (Q26–Q28) employed a four-point scale. Score percentages ranged from 0 to 100% in questions 25, 29, and 35. The study did not include the questions Q25 and Q29 because they were not determine the perception of nurses on hand hygiene. Five questions Q30– Q34 were ranked on a seven-point scale. Question Q30 has eight sub-questions on a seven-point scale. Question Q35 was assessed independently for self-reported HH performance, with score percentages ranging from 0 to 100%.

### Sample and sampling technique

The sample size for this study was calculated using two independent mean computations, with the study’s power set at 80%, type one error set at 0.05, the mean ratio set at one, and the standard deviation of knowledge scores from a prior study set at 0.11 [22]. The minimum sample size required was 424, and the total number of samples required was 530, with a 20% dropout rate factored in.

A method of quota sampling was used. The first quota consisted of four hospitals. The distribution of survey participants from the sample size was randomised and proportionate to the list of qualified nurses in each department.

Registered nurses with at least six months of experience working at tertiary hospitals participated in our study. Nurses who worked solely as administrative assistants were excluded from the study. Nurses who self-reported their performance were defined as estimating how much HH was done in accordance with HH recommendations [23]. The knowledge score, perception score, and self-reported HH performance score were all evaluated using the variables specified.

### Data collection

We acquired informed consent from all the participants who were chosen based on our research design. All information would be used solely for research purposes and would be kept in strict confidence. Questionnaires were given to nurses following coordination with hospitals, and each nurse had 10 minutes to complete the questionnaire before it was collected. The recruitment method includes keeping participants’ information until the research is finished. Each participant was assigned a code number.

### Statistical analysis

All 530 questionnaires, numbered 1 through 530, were distributed to the participants. The data was entered into Microsoft Excel Office 365 for analysis and then exported to IBM SPSS version 26. After the data was entered, it was processed, validated, and cleaned. A provisional data summary was utilized to find missing values and look for errors. Data for missing sample objects that were unique and specific were removed from the analysis. To find correlations between knowledge score, perception score, and HCAIs Pre and Post assessment, researchers used a Pearson correlation analysis. Statistical significance was defined as a two-tailed and P value of less than 0.05.

### Ethical consideration

Ethical approval was obtained from the Universiti Sains Malaysia Human Etiquette Committee with reference number: USM/JEPeM/19100595 and from the National Medical Research Registry with reference number: NMRR-19-3365-51286. The data were strictly limited, and access was only given to the author and supervisor. Analyzes and publications were consequently rendered without the identities of the selected participants.

## Results

### Socio-demographic and work characteristics of participants

A total of 530 questionnaire surveys were distributed, with 438 fully completed questionnaires yielding an 82.6 percent response rate. Nurses were on average 38.4 (7.314) years old, and 93.2 percent of them were female. The majority of the participants lacked a post-basic diploma. The average clinical practice experience of the participants was 14.93 (6.913) years. Almost every participant in the study worked as a staff nurse (90.6%) or a non-infection care nurse (95.2%). Surprisingly, 91.8 percent of participants stated they had had formal HH training in the past three years, and 97.5 percent reported they used an alcohol-based cleaner on a daily basis for HH. Table 1 provides a summary of the participants’ socio-demographic and work characteristics.

**Table 1:** Socio-demographic and work characteristics of participants (*n* = 438)

Variables	n (%)	Mean(±SD)
Gender		
Female	408(93.2)	
Male	30(6.8)	
Age		38.43±7.314
Marital status		
Married Unmarried	393(89.7) 45(10.3)	
Education level		
O level 3-Year college/Diploma Master/PhD Diploma with post-basic	24(5.5) 353(80.6) 5(1.1) 56(12.8)	
Working experience		14.93±6.913
Infection control nurse (ICN)		
ICN Non-ICN	21(4.8) 417(95.2)	
Positions		
Nurse Sister (charge nurse) Matron (head nurse)	397(90.6) 36(8.2) 5(1.1)	
Received HH Training within the last three years		
Yes No	402(91.8) 36(8.2)	
Routinely use hand rub for HH		
Yes No	427(97.5) 11(2.5)	

### Knowledge, perception and self-reported HH performance scores

The mean knowledge score among nurses in Kelantan’s tertiary care hospitals was 15.08±1.960, with a range of 0 to 25. Perception participants’ mean score toward HH was 68.02±10.144, with a range of 0 to 81. Furthermore, with a range of 0 to 100, the average self-reported HH performance in percentage was 87.58±12.025. Table 2 shows the participants’ knowledge, perception, and self-reported HH performance scores

**Table 2:** Result of knowledge, perception, and self-reported performance scores on HH among nurses at tertiary care hospitals in Kelantan, Malaysia. (*n* = 438)

Variables	Range	Mean(±SD)
Knowledge	8 – 22	15.08(±1.960)
Perception	36 – 81	68.02(±10.144)
Self-reported HH performance	34 – 100	87.58(±12.025)

### Correlation of knowledge, perception and self-reported HH performance scores

A Pearson’s correlation analysis in Table 3 showed significant positive correlations between knowledge and perception, r(436)=0.17, *p*<0.001. Perception and self-reported HH performance showed a positive correlation, r(436)=0.27, *p*<0.001. These findings indicated that knowledge scores did not show significant correlations with self-reported HH performance.

**Table 3:** Result of correlation knowledge, perception, and self-reported performance scores on HH among nurses at tertiary care hospitals in Kelantan, Malaysia (*n* = 438)

Variables	Knowledge	Perception	Self-reported HH performance
Knowledge	1.00		
Perception	0.17**	1.00	
Self-reported HH performance	0.06	0.27**	1.00

** *P*<0.001: Two tailed by Pearson’s correlation analysis

Correlation of Knowledge and Perception scores with Health Care Associated infections in Hospital

Table 4 shows significant positive correlations between knowledge scores and HCAIs Pre assessment r(435)=0.15, *p*<0.001 and HCAIs Post assessment, r(435)=0.13, *p*<0.001. The finding, in other hand indicated that perception scores did not show significant correlations with HCAIs Pre assessment and Post assessment.

**Table 4:** Result of correlation knowledge and perception scores with health care associated infections in tertiary care hospitals in Kelantan, Malaysia. (*n* = 438)

Variables	Knowledge	Perception	HCAIs Pre assessment	HCAIs post assessment
Knowledge	1.00			
Perception	0.17**	1.00		
HCAIs Pre assessment	0.15**	0.32	1.00	
HCAIs Post assessment	0.13**	0.01	0.974**	1.00

** *P*<0.001: Two tailed by Pearson’s correlation analysis

## Discussion

The majority of the respondents in this survey were female, which matches previous research in Sibu, Malaysia [13], suggesting that the nursing career in Malaysia is naturally dominated by women. The average age of the nurses was higher than previous surveys of nurses, which found average ages of 31.2 ±7.3 years [19], 32.7±4.6 years [24], and 29.4±5 years [25] suggesting that the majority of the nurses in the hospital were young workforce. More clinical career experience had a larger effect on HH practice [26], which was attributed to respondents’ age. Participants had an average work experience which was better than prior research including 12.22 ±7 years [27], 14.2 ±10.2 years [28], and 10.12 ±13.50 years [29]. Almost all nurses, have earned HH training in the previous three years, relative to lower figures in other surveys [18, 30], indicating that the infection prevention unit and top management of the hospitals introduced a strong training programmed coverage for nurses.

The mean knowledge score was lower than previous studies that showed knowledge scores of 17.6±2.5 [19], 15.52±4.8 [31], and 19.5± 2.3 [32] by using the same tool. The variability of our findings is probably due to differences in gender, work experience and had received HH Training. According to Asadollahi, Arshadi Bostanabad [33], lack of nurses knowledge of hand hygiene related to work experience, type of employment and previous training of hand hygiene. Another study by Al-Mohaithef, Chandramohan [34] gender, work experience and had training course all had a significant effect on the knowledge score. Despite the fact that almost all of our respondents received training in the last three years, their knowledge does not reflect the study’s findings. In other words, these programmed have had little effect on improving nurses’ knowledge in this area. We should reevaluate the effectiveness of HH training courses, educational method and enhance our technique of training program instruction based on the knowledge score’s findings. The need of reevaluating the training curriculum and method is stressed by Zakeri, Ahmadi [22] and Asadollahi, Arshadi Bostanabad [33]. An outstanding combination of approach training programs for inspiring involvement that are more effective at integrating cognitive, social, and therapeutic approaches [35].

The current perception score finding appears to be consistent with previous research that discovered a higher perception score [19, 36, 37]. These findings show that nurses have a positive perception of HH, which includes the value of HH to (self, manager, patient, and colleagues), the role of the organization in HH, various measures to improve HH in the institution, HH having a preventive effect, and reducing HCAIs patient outcome. Vikke and Vittinghus [23] found similar results in a previous study. An idea, a belief, or the way someone thinks and feels about something is defined as perception. When it came to the impact of HCAIs, the majority of nurses believed that infection could be avoided by using HH [30].

Previous studies appear to be consistent with the self-reported HH performance score. This result was higher than the previous study by Oh [25], but lower than the previous study by Vikke, Vittinghus [23]. Nurses self-reported their HH performance and estimated how frequently they performed HH as recommended. As a result, nurse performance may be related to a number of factors, including motivation, the role of a peer or higher-ranking staff member in the room when performing HH, and the nurses’ attitude. How do HCWs react when colleagues or superiors refer to them as a role model? According to Oyapero and Oyapero [31], the superior had an impact on their decision to conduct HH. According to Lankford and Zembower [32], a role model (peer hand-compliance) and group performance practises HH can influence hand-hygiene behaviours and performance. As a result, the attitudes of HCW role models and nurses were linked to HH self-reported performance [11]. Another reason is that nurses may overestimate their actual HH performance because they are anxious to be observed by senior staff or management [20].

Correlation study demonstrated a favourable association between knowledge and perception, comparable to studies by Al-khawaldeh, Al-Hussami [38] and Erasmus, Otto [39]. The impact of HCAIs may be influenced by the nurses’ degree of knowledge on HH, according to an Erasmus study that employed an extended theory of planned behaviour model to describe the nurses’ perceptions of risk of breaking HH rules and the impact of HCAIs [39]. These findings, on the other hand, contradict those of researches [19, 32], which revealed no strong connection. Perceptions and self-reported HH performance have a significant correlation, according to the findings, which are similar to previous research [15, 19, 32, 38]. As a result of the improved perception, particularly the perception of being a role model for other co-workers, hand hygiene compliance increased [20]. We should stress being a role model and monitoring starting from stakeholder and leader nurses to increase the intention to adhere to hand hygiene. Furthermore, no significant correlations were found between knowledge scores and self-reported HH performance, demonstrating that having more knowledge does not always reflect better hand washing habits. Inadequate hand-washing knowledge, on the other hand, is a factor that can negatively affect hand-washing behavior [38].

The presence of HCAIs is an important indicator of patient safety and quality of care. Infection-control behaviours are mediated not only by rational knowledge or motivation, but also by social, emotional, and frequently stereotyped behaviors [37]. In the current finding, a positive relationship between knowledge and HCAIs pre and post assessment in the hospital appears. Despite the low degree of association, knowledge remains an important component associated with hand hygiene performance, which will have a significant impact on HCAI mortality and morbidity in hospitals. Compliant is a multifaceted issue that necessitates both knowledge and behaviour. Educational awareness and regular reminders are required to maintain high rates of hand hygiene performance [38]. Perception does not appear to correlate well with HCAIs pre and post hospital assessment. This contradicts previous findings by McClung and Obasi [39]. HCWs’ perceptions of patient safety and clinical consequences are motivating factors for lowering HCAIs.

### Limitations

The current study used a cross-sectional design. It limits the finding's ability to make a causal inference. As a result, a better study design, such as an intervention design with a randomized controlled group, is advised. Aside from that, using a self-administered questionnaire usually results in a low response rate. To address this issue, we collaborate closely with the nursing unit to collect respondents. We made sure that the respondents understood the purpose of the study before they filled out the questionnaire.

## Conclusion

The importance of monitoring and evaluating nurses' knowledge, perceptions, and performance as a reference point for all stakeholders to assess the efficacy of the HH strategy was demonstrated in this study. Nurses at Kelantan's tertiary care institutions had a higher overall perception and self-reported HH performance than in earlier studies. The most obvious finding was that knowledge scores were lower than in earlier trials. Poor HH practises could result from a lack of knowledge about HH during patient care, leading to an increase in HCAIs and AMR. The findings of this study point to a critical reevaluation of the effectiveness of the HH training course, as well as an improvement in our training programme. We believe that applying WHO's comprehensive multimodal HH, which includes improvement techniques ranging from one to five phases and considering future research at a national level, is the key to improving HH performance among HCWs.

## Data Availability

The datasets generated and/or analyzed during the current study are not publicly available due to the need to maintain the anonymity of participants and the confidentiality of the data. However, the datasets are available from the corresponding author on reasonable request.

## Abbreviations

HH: Hand hygiene
HCW: Health care worker
HCAIs: Health care associated infections
AMR: Antimicrobial resistance
